# A Prognosis Marker SLC2A3 Correlates With EMT and Immune Signature in Colorectal Cancer

**DOI:** 10.3389/fonc.2021.638099

**Published:** 2021-06-15

**Authors:** Huabin Gao, Jiangtao Liang, Jing Duan, Lin Chen, Hui Li, Tiantian Zhen, Fenfen Zhang, Yu Dong, Huijuan Shi, Anjia Han

**Affiliations:** Department of Pathology, The First Affiliated Hospital, Sun Yat-Sen University, Guangzhou, China

**Keywords:** SLC2A3, colorectal cancer, epithelial–mesenchymal transition, PD-L1, prognosis marker

## Abstract

SLC2A3 is a membrane transporter that belongs to the solute carrier family, whose function includes transmembrane transport and glucose transmembrane transport activity. To clarify the expression and role of SLC2A3 in colorectal cancer (CRC), we analyzed the TCGA and GEO databases and found that SLC2A3 mRNA levels were significantly higher in CRC tissues than that in adjacent non-tumor tissues. Furthermore, high expression of SLC2A3 predicted poor overall survival and disease free survival for CRC patients. For validation, we collected 174 CRC samples and found that SLC2A3 expression was higher in CRC tissues than that in adjacent non-tumor colorectal mucosa tissues by immunohistochemistry staining. Further study showed that high expression of SLC2A3 was enriched in epithelial–mesenchymal transition (EMT) classical pathway, interferon-γ pathway by GSEA analysis enrichment, indicating that SLC2A3 may play a key role in the progression of CRC through EMT and immune response, which also has been validated by the global gene expression profiling of human CRC cell lines. The expression of SLC2A3 was positively correlated with CD4 and CD8+T cells by using TIMER and EPIC algorithm, respectively. SLC2A3 knockdown suppressed migration and inhibited the expression of Vimentin and MMP9 in CRC cell line SW480 and RKO. Meanwhile, PD-L1 expression was also significantly attenuated in SW480 and RKO cells transfected with SLC2A3 siRNA. The result suggests that SLC2A3 may be involved in the immune response of CRC by regulating PD-L1 immune checkpoint. In our series, SLC2A3 and PD-L1 positive expression was 74% (128/174) and 22% (39/174) of CRC, respectively. SLC2A3 expression was significantly associated with perineural invasion in CRC patients. In conclusion, SLC2A3 may play an important role in progression of CRC by regulating EMT and PD-L1 mediated immune responses.

## Background

Colorectal cancer (CRC) is a leading malignant tumor in the digestive tract (1). At the time of diagnosis about 30% of CRC patients had distant metastases, while about 20% of CRC patient had recurring and distant metastasis in 5 years after radical resection (2). Guinney et al. divided colorectal cancer into four distinct molecular subtypes (CMS) by gene expression profiling analysis: CMS1 represented microsatellite unstable immune type, accounting for about 14%, which had high gene mutation, microsatellite instability and strong immune activation. CMS2 represents the typical Wnt and Myc signaling pathways, accounting for about 37%. CMS3 represents metabolic type, accounting for about 13%, epithelialization accompanied by significant metabolic disorder. CMS4 represents the mesenchymal type, accounting for 23%, with significant transformation growth factor activation, interstitial infiltration, and angiogenesis (3). CMS classification may be a prognostic and predictive factor for CRC.

The SLC2A family includes 14 members from SLC2A1–SLC2A14, known as glucose transporters, that enables nutrients and glucose to pass through the hydrophobic cell membrane (4). The expression of these members is tissue specific. For example, SLC2A1 is generally expressed in all tissues, while SLC2A2 is expressed in liver tissues and SLC2A3 is expressed in brain tissues (5). Recent studies have indicated that SLC2A family increased in different tumors, which exhibit the potential oncogenic effect of SLC2A family. SLC2A1 is boost in CRC tissues and predicts poor prognosis and clinical characteristics (6), SLC2A2 is highly expressed in hepatocellular carcinoma (7). SLC2A3 expression is up-regulated in invasive gliomas, and high expression indicates poor prognosis (8). Recent reports revealed that SLC2A3 may take part in the metastasis of breast cancer cell to the brain (9). However, the role of SLC2A3 in CRC has not been well clarified.

In this study, we explored the expression of SLC2A3 in CRC by TCGA database and analyzed the relationship between SLC2A3 expression and prognosis and other clinical features. Furthermore, we detected SLC2A3 expression in our CRC samples and clarified the underlying mechanism. Our study suggests that SLC2A3 could be used as a prognosis marker in CRC and promotes the progression of CRC through EMT and PD-L1.

## Material and Methods

### Data Mining

TCGA CRC gene expression RNA-seq data and related clinical phenotype were downloaded from the UCSC website (10). There were 380 cases of CRC tissue and 51 cases of adjacent non-tumor tissue. To investigate the underlying mechanism of SLC2A3 expression, we divided them into low-SLC2A3 expression group and high-SLC2A3 expression group based on SLC2A3 median expression level. Moreover, GSE17536(N = 177) and GSE17537 (N = 55) data (11–14) were applied for further prognosis validation, which were from Gene Expression Omnibus (GEO) data (https://www.ncbi.nlm.nih.gov/geo/query/acc.cgi?acc=GSE17536 and https://www.ncbi.nlm.nih.gov/geo/query/acc.cgi?acc=GSE17537). Among them, 55 CRC samples were from Vanderbilt Medical Center (VMC), while 177 CRC samples from the Moffitt Cancer Center. Human CRC cell lines expression profile were downloaded from GSE59857 (15, 16), which included 155 established CRC cell lines and two human fetal intestine cell lines.

### Gene Set Enrichment Analysis

GSEA software (v4.0.3) (17, 18) was carried out to explore the mechanisms of SLC2A3 expression on the progression of CRC. HALLMARK gene set was obtained from MSigDB database V7.2. The Nominal p-value (NOM p <0.05) was considered to be significantly enriched.

### Immune Cell Infiltration Analysis

The abundance of CRC immune cell infiltration was obtained from TIMER (19, 20). The TIMER web server is a comprehensive resource for systematical analysis of immune infiltrates across diverse cancer based on deconvolution. The composition of six immune infiltrates (B cells, CD4+ T cells, CD8+ T cells, Neutrophils, Macrophages, and Dendritic cells) was predicted by input the gene expression profile data of tumor samples. EPIC (21) method was used to validate the results. EPIC is an analytical method to access the proportion of Immune and cancer cells from bulk tumor gene expression data.

### Patients and Specimens

A total of 174 paired paraffin-embedded CRC specimens and corresponding adjacent non-tumor colorectal mucosal tissues were obtained from our Department of Pathology, the first Affiliated Hospital, Sun Yat-Sen University, Guangzhou, China, between January 2013 and December 2013. No patients had received chemotherapy and/or radiotherapy before operation. The histopathology of the disease was determined by two pathologists according to the criteria of the World Health Organization. Prior patient consent and approval were obtained from the Institutional Research Ethics Committee. Tissue microarray was constructed by 1.5-mm cores.

### Immunohistochemistry Staining and Evaluation

Immunohistochemistry staining was performed as we previously described (22), where the working concentration of SLC2A3 antibody(20403-1-AP, Proteintech, Chicago) was 1:200. Immunohistochemical staining was assessed and scored by two independent pathologists. Staining intensity was graded as: absent staining as 0, weak as 1, moderate as 2, and strong as 3. The percentage of stained cells was categorized as positive cancer cells/total cancer cells. The staining score for each tissue was calculated by the area score × the intensity score. PD-L1 antibody(DAKO 22C3, Denmark)was performed according to the manufacturer’s guidance. The working concentration was 1:50. PD-L1 score is based on combined positive score (CPS), which is simply the percentage of living cancer cells, cancer associated lymphocytes and macrophages stained with partial or complete membranes at any intensity, namely the number of PD-L1 positive cells/all living tumor cells × 100. According to the receiver operative characteristic (ROC) analysis, the optimal cutoff value of SLC2A3 and PD-L1 was a staining score of 0.175 and 0.025 or lower defied as low-SLC2A3 and low-PD-L1 group respectively, while, a staining score of 0.175 and 0.025 higher defied as the high-SLC2A3 and high-PD-L1 group, respectively.

### Cell Culture and siRNA Transfection

SW480 and RKO cells were purchased from Shanghai cell bank (Shanghai, China) and cultured in 10% FBS+DMEM. SLC2A3 siRNA and negative control NC were purchased from RiboBio Guangzhou (China). The targeted sequences are followed si-SLC2A3: GTAGCTAAGTCGGTTGAAA. siRNA transfection was performed by using Lipofectamine3000 reagent (Thermo Fisher Scientific)according to manufacturer’s protocols.

### Quantitative Real Time PCR

The total RNA in cells was extracted with trizol reagent (Invitrogen, USA), and the relative mRNA expression was normalized to the GAPDH. The following primer sequences are purchased from SANGON BIOTECH (Shanghai, China) SLC2A3-Forward: GGTCGCTTGGTTATTGGC, SLC2A3-Reverse: ACCGCT GGAGGATCTGCT. Quantitative real time PCR was performed as our previously described (23).

### Western Blot

Cells were collected after siRNA transfection for 48 h. The antibodies include SLC2A3 primary antibody (Protein tech #20403-1-AP), Vimentin (CST#5741), MMP9 (CST#13667), and PD-L1 (CST#13684). The working concentrations of the above primary bodies are 1:1,000. The working concentrations of anti-rabbit IgG, HRP-linked antibody (CST#7074) is 1:4,000. GAPDH (CST #5174) was used as loading control.

### Migration and Invasion Assays

The migration experiment was conducted by first laying SW480 and RKO cells in a 6-well plate and transfecting them with siRNA for 48 h, then laying 10^5^ cells into 100 ul serum-free DMEM in the upper compartment, adding 500 ul 20% FBS to the lower compartment, and collecting the compartment after 48 (RKO) and 60 h (SW480), respectively. For the invasion experiment, 50 ul matrix glue (1:8) was first placed in the upper chamber, and the chamber was collected after 48 (RKO) and 84 h (SW480), respectively. All the cells were collected and fixed with 4% paraformaldehyde and stained with 0.1% crystal violet. The membrane of the cells was cut off and scanned for statistics. Five high-power (40×) field counts were randomly selected for each membrane.

### Statistics Analysis

SPSS 25.0 statistical software and GraphPad Prism 8 (GraphPad Software) were used for statistical analysis. Unpaired, two tailed Student’s test was used to compare data between the two groups. Chi-square test and rank sum test are used to compare different parameters. Spearman correlation analysis was used to evaluate correlations between variables. Univariate and multivariate logistic regression analysis were used to analyze the relationship between SLC2A3 expression levels and clinical characteristics. P<0.05 was considered to be statistically significant.

## Results

### SLC2A3 Serves as an Oncogenic Role in CRC and High SLC2A3 Expression Predicts Poor Prognosis

**Figure 1:**
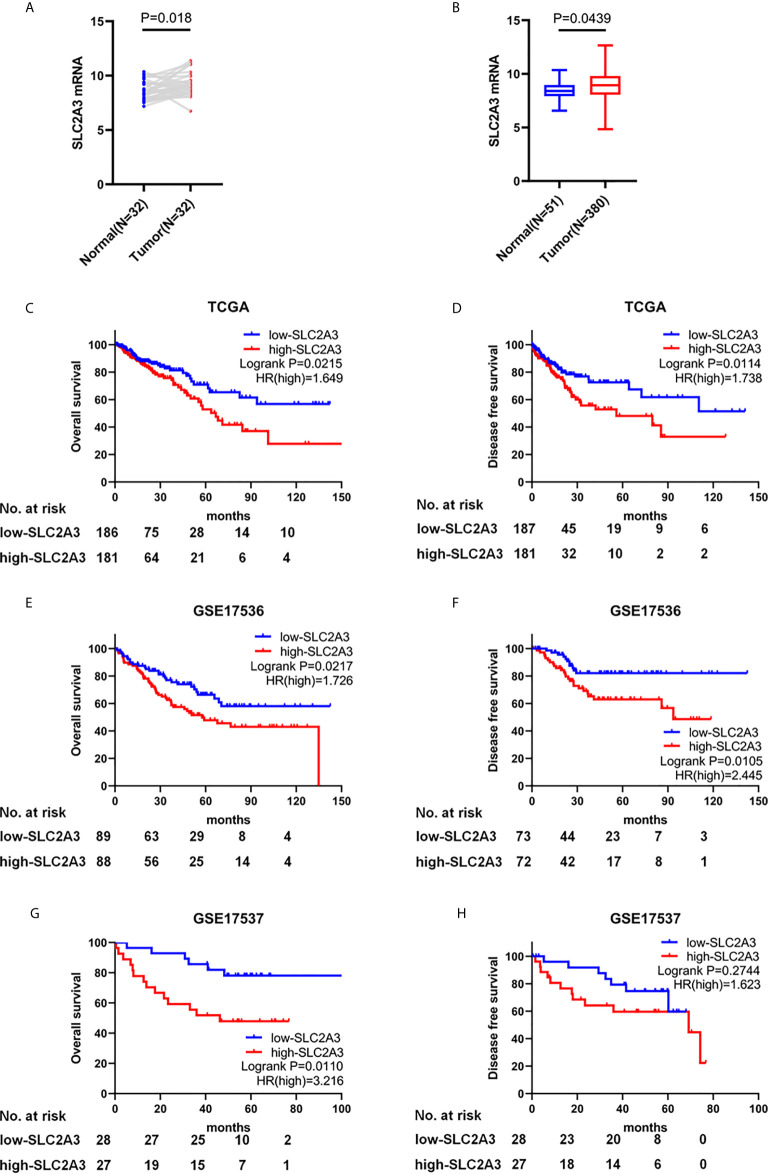
SLC2A3 serves as an oncogenic role in CRC and high SLC2A3 expression predicts poor prognosis. **(A)** The expression of SLC2A3 in paired CRC samples from TCGA. **(B)** The expression of SLC2A3 in all CRC samples from TCGA. The correlation between SLC2A3 expression and survival status in **(C, D)** TCGA, **(E, F)** GSE17536, and **(G, H)** GSE17537.

To investigate the expression level and prognosis role of SLC2A3 in CRC, we analyzed the RNA-seq datasets and corresponding clinical features from TCGA CRC database and found that SLC2A3 was significantly up-regulated in paired CRC tissues (N = 32) compared with adjacent normal tissues (**Figure 1A**). We obtained the same result when compared all CRC tissues and normal tissues (**Figure 1B**). CRC patients with SLC2A3 high expression exhibited poor overall survival and disease free survival (**Figures 1C, D**). We further studied the relationship between SLC2A3 expression and prognosis from GSE17536 and GSE17537 data (**Figures 1E–H**). Consistent with TCGA results, the GEO results showed that up-regulated SLC2A3 expression was correlated with poor prognosis in CRC. The results indicate that SLC2A3 could be a prognosis marker for CRC patients.

### Relationship Between SLC2A3 Expression and Clinical Features of CRC

**Figure 2:**
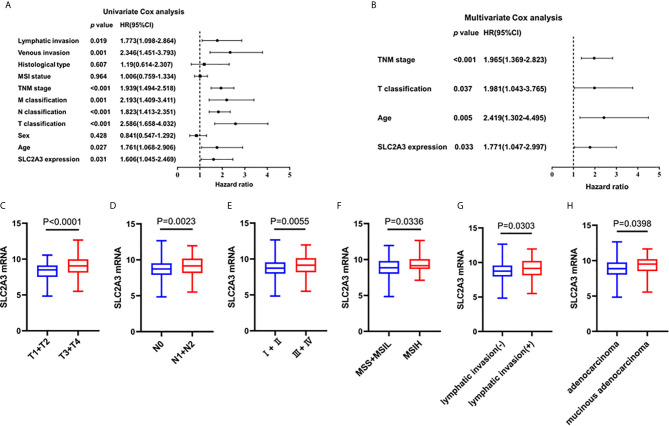
SLC2A3 expression was associated with clinicopathological features of CRC based on TCGA. **(A)** Univariate and **(B)** multivariate Cox analysis of SLC2A3 expression and clinicopathological variables. SLC2A3 expression associated with **(C)** increased invasive depth, **(D)** lymph node metastasis, **(E)** higher stage, **(F)** MSI-H, **(G)** lymphatic invasion, and **(H)** mucinous adenocarcinoma.

To explore the relationship between SLC2A3 expression and clinical features of CRC, the results showed that SLC2A3 expression was significantly associated with T classification, N classification, TNM stage, MSI status, histological type and lymphatic invasion (**Table 1**). Univariate COX regression analysis showed that SLC2A3 expression, age, T classification, N classification, M classification, TNM stage, histological type, venous invasion and lymphatic invasion were prognostic risk factors (**Figure 2A**). Multivariate COX regression analysis indicated that SLC2A3, age, T classification and TNM stage were independent prognostic risk factors (**Figure 2B**). Moreover, SLC2A3 expression was significant up-regulated in T3 + T4 groups (**Figure 2C**), N1 + N2 groups (**Figure 2D**), TNM III + IV groups (**Figure 2E**), MSI-H groups (**Figure 2F**), lymphatic invasion groups (**Figure 2G**) and mucous adenocarcinoma groups (**Figure 2H**). The results suggest that high SLC2A3 expression is associated with more aggressive behavior of CRC.

**Table 1:** Relationship between SLC2A3 expression and clinicopathological features of CRC in TCGA.

Characteristics	low SLC2A3	high SLC2A3	P value
	No. (%)	No. (%)	
Age (years)			0.428
Mean year	64 mean	65 mean	
<60	69 (53.1)	61 (46.9)	
≥60	120 (48.8)	126 (51.2)	
Sex			0.295
Male	99 (47.8)	108 (52.2)	
Female	90 (53.3)	79 (46.7)	
T classification			0.007
T1	9 (90)	1 (10)	
T2	36 (63.2)	21 (36.8)	
T3	121 (46.9)	137 (53.1)	
T4	22 (44.9)	27 (55.1)	
N classification			0.004
N0	118 (57.3)	88 (42.7)	
N1	44 (44.4)	55 (55.6)	
N2	27 (39.7)	41 (60.3)	
M classification			0.483
M0	132 (51.8)	123 (48.2)	
M1	55 (47.8)	60 (52.2)	
TNM stage			0.01
I	37 (64.9)	20 (35.1)	
II	71 (52.2)	65 (47.8)	
III	47 (41.6)	66 (58.4)	
IV	24 (46.2)	28 (53.8)	
MSI statue			0.026
MSS	136 (53.5)	118 (46.5)	
MSI-L	28 (44.4)	35 (55.6)	
MSI-H	20 (37.7)	33 (62.3)	
Histological type			0.043
adenocarcinoma	172 (52.3)	157 (47.7)	
mucinous adenocarcinoma	15 (35.7)	27 (64.3)	
Venous invasion			0.17
absent	130 (52.4)	118 (47.6)	
present	33 (43.4)	43 (56.6)	
Lymphatic invasion			0.001
absent	127 (55.7)	101 (44.3)	
present	36 (35.3)	66 (64.7)	

### SLC2A3 Regulates the Progression of CRC Through EMT Pathway

**Figure 3:**
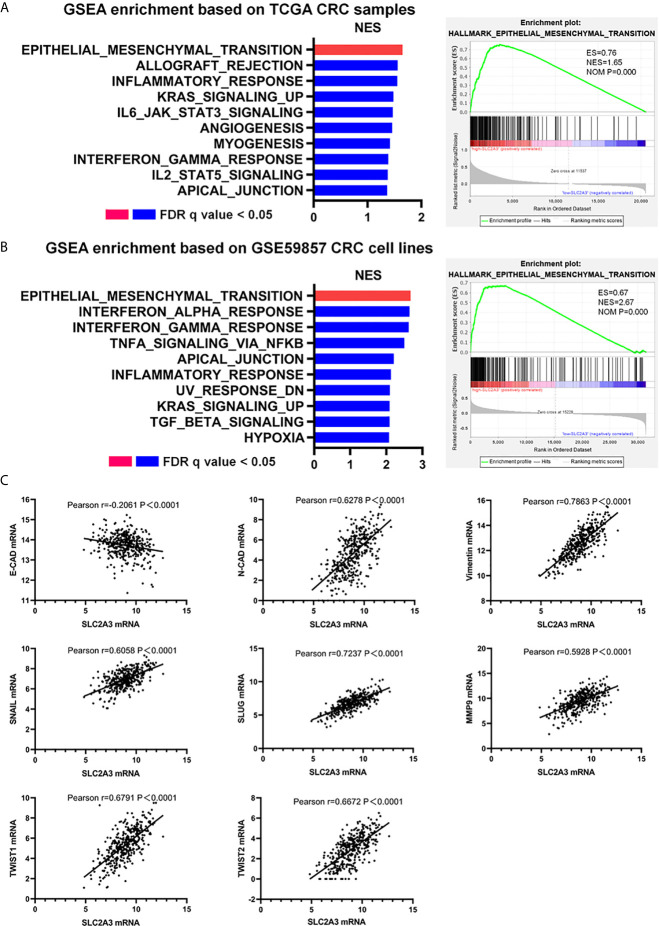
SLC2A3 expression correlated with EMT signatures in CRC. **(A)** GSEA enrichment based on TCGA CRC samples, **(B)** GSEA enrichment based on GSE59857 CRC cell lines, and **(C)** TCGA database showed SLC2A3 expression was positively correlated with N-CAD, Vimentin, SNAIL, SLUG, MMP9, TWIST1, and TWIST2 expression, negatively correlated with E-CAD expression. All gene sets were significantly enriched at nominal p value <0.05 and FDR q value <0.05. NES, normalized enrichment score. FDR, false discovery rate.

To clarify the underlying mechanism of SLC2A3 in the progression of CRC, we performed GSEA enrichment based on high-SLC2A3 expression and low-SLC2A3 expression group. HALLMARK EMT pathway was the top enriched gene signature when compared high-SLC2A3 and low-SLC2A3 expression group from TCGA CRC samples (**Figure 3A**). To verify the conclusion, we analyzed CRC cell lines expression from GSE59857. In line with TCGA CRC samples, high-SLC2A3 group was also enriched in the EMT pathway (**Figure 3B**). Additionally, there was a positive correlation between SLC2A3 expression and N-CAD and Vimentin expression, a negative correlation with E-CAD expression. The consistent result was observed by other EMT classical signatures including SNAIL, SLUG, MMP9, TWIST1, and TWIST2 (**Figure 3C**). These findings suggest that SLC2A3 might promote CRC progression by regulating EMT pathway.

### SLC2A3 Regulates the Progression of CRC Through Immune Response

**Figure 4:**
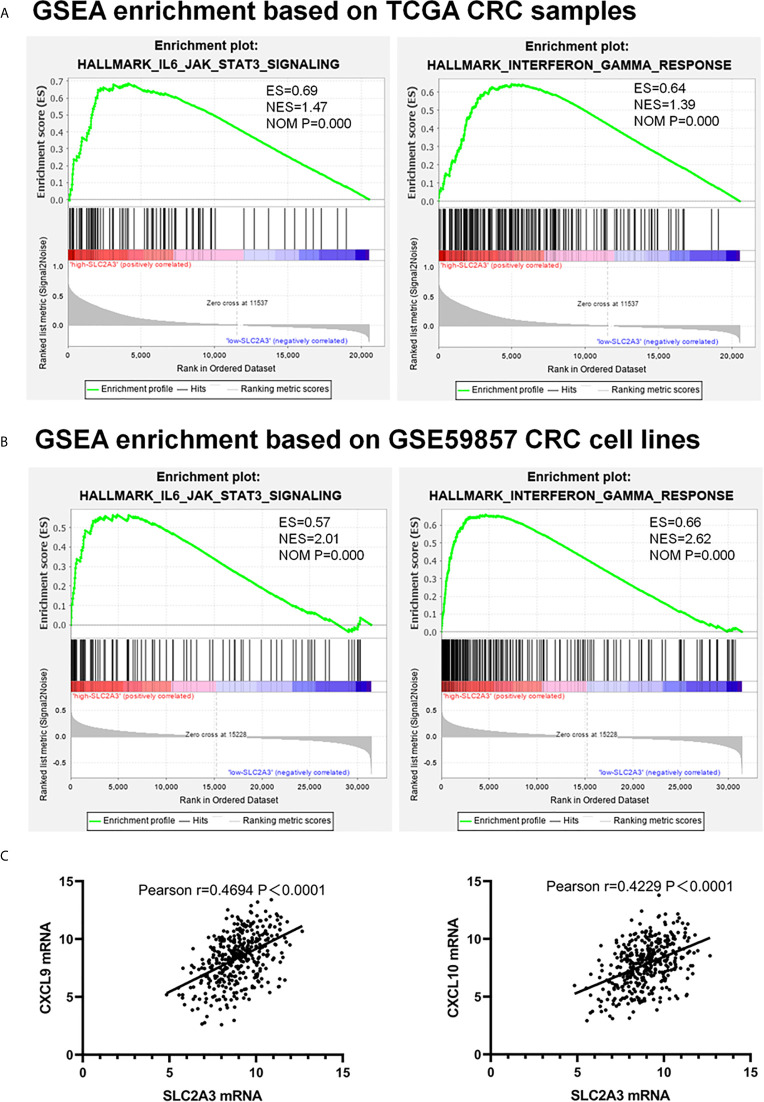
SLC2A3 expression correlated with IL6/JAK/STAT3 signaling and interferon-γ hallmark gene sets. **(A)** TCGA database, **(B)** GSE59857 database, **(C)** the positive correlation between SLC2A3 expression and CXCL9 and CXCL10 expression based on TCGA.

Recent immune response and immune environment regulates tumor progression (24). To clarify whether SLC2A3 has an impact on immune response, we analyzed GSEA results of TCGA (**Figure 4A**) and GSE59857 (**Figure 4B**) database and reached a consistent conclusion that high SLC2A3 expression group was enriched in the inflammatory response pathway including the IL6/JAK/STAT3 signaling pathway and the interferon-γ response pathway, respectively. Furthermore, both enrichment of immune signature of CRC samples and CRC cell lines showed that SLC2A3 expression was significantly related to immune response gene expression including CXCL9 and CXCL10 (**Figure 4C**).

### High Expression of SLC2A3 Increased PD-L1 Expression in CRC

**Figure 5:**
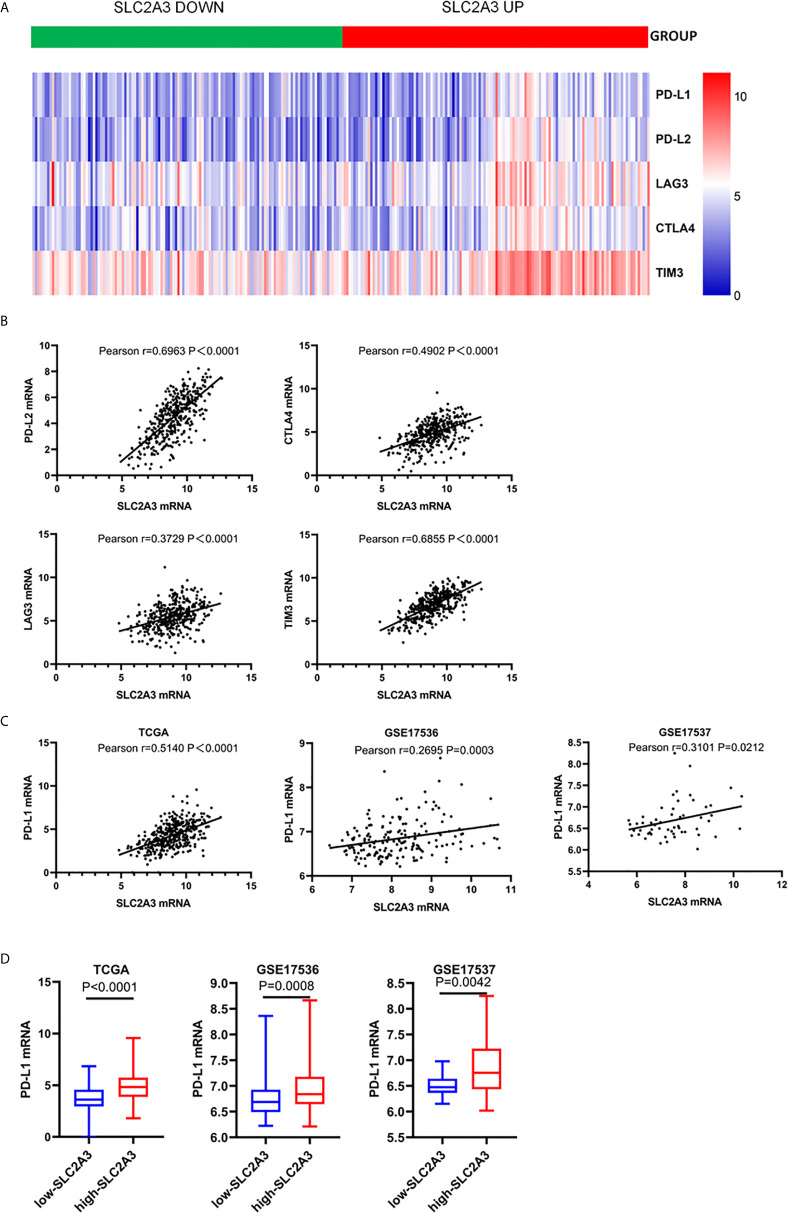
SLC2A3 expression was significantly associated with PD-L1 expression. **(A)** heatmap of immune checkpoints based on SLC2A3 expression; **(B)** the correlation between SLC2A3 expression and immune checkpoints; **(C)** the correlation between SLC2A3 expression and PD-L1 expression in different database; **(D)** PD-L1 mRNA expression increased by SLC2A3 upregulation.

Since cancer cells also drive the expression of PD-L1 mRNA *via* the IFN-/JAK/STAT1 signaling pathway (25). we hypothesized that SLC2A3 may be associated with immunotherapy checkpoint genes such as PD-L1. Heatmap and correlations between SLC2A3 and immune checkpoints including PD-L1, PD-L2, LAG3, CTLA4, and TIM3 were exhibited (**Figures 5A, B**). Significant positive correlation between SLC2A3 and PD-L1 expression was observed in CRC samples of TCGA, GSE17536 and GSE17537 databases (**Figure 5C**). Upregulation of SLC2A3 mRNA level also correlated with increased PD-L1 mRNA level based on the above three databases (**Figure 5D**).

**Figure 6:**
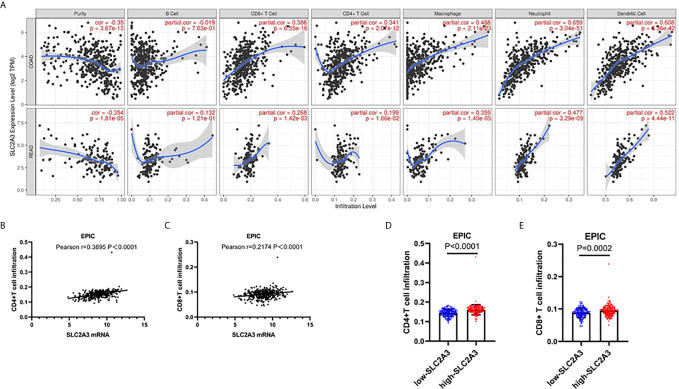
SLC2A3 significantly correlated with T cell infiltration in CRC based on **(A)** TIMER and **(B, C)** EPIC algorithm; **(D, E)** SLC2A3 expression increased CD4+ and CD8+T cell infiltration in CRC based on EPIC algorithm.

Notably, recent studies revealed that tumor-Infiltrating Lymphocytes (TILs) in CRC tumor bed have been interrelated with favorable outcome (26), we were curious about the effect of SLC2A3 expression on TILs in CRC. Using online analysis TIMER, we found that SLC2A3 was significantly correlated with neutrophils, dendritic cells, macrophages, CD4+T cells and CD8+T cells (**Figure 6A**). In accordance with TIMER results, EPIC results showed that SLC2A3 mRNA expression was significantly correlated with CD4+T cells and CD8+T cell infiltration (**Figures 6B, C**). SLC2A3 upregulation was correlated with increased CD4+T cells and CD8+T cell infiltration, respectively (**Figures 6D, E**). Together, our result revealed that SLC2A3 might be as a novel predictor of immunotherapy response in CRC.

### SLC2A3 Expression in Our CRC Samples and Regulates EMT Signaling Pathway and PD-L1 Expression

**Figure 7:**
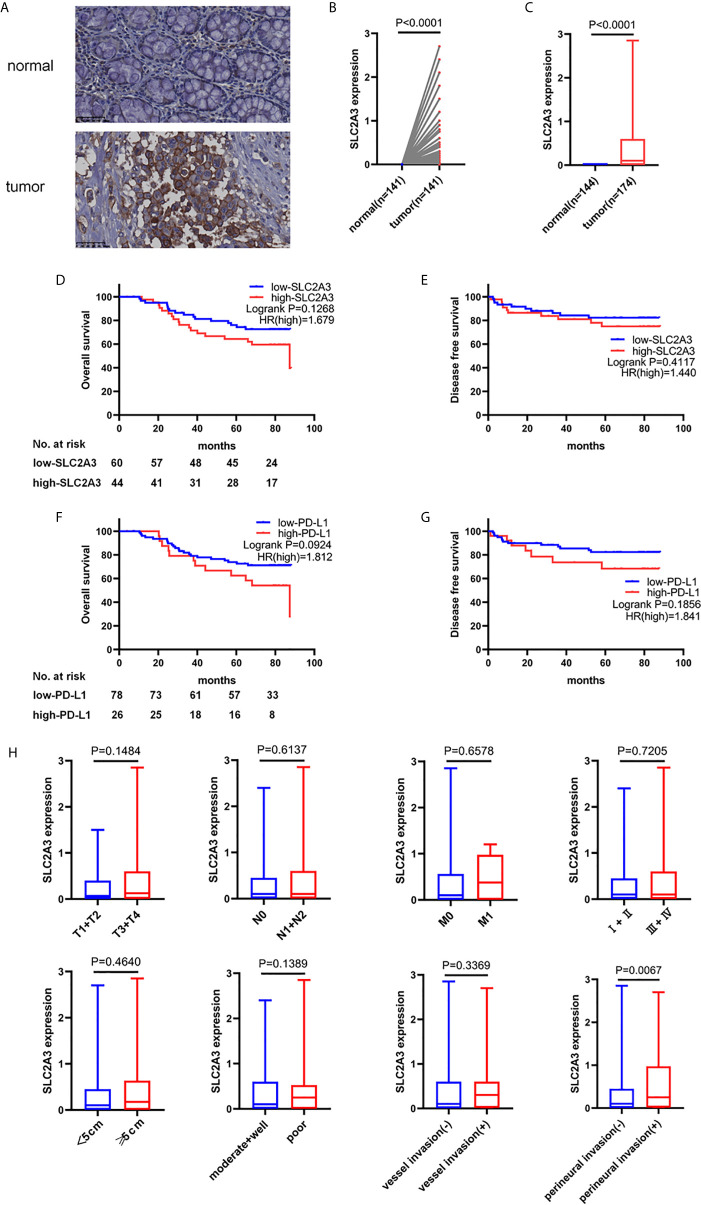
SLC2A3 and PD-L1 expression in CRC tissues. **(A)** SLC2A3 expression in CRC and adjacent non-tumor colorectal mucosal tissue by immunohistochemistry staining. Expression of SLC2A3 in **(B)** paired and **(C)** unpaired CRC tissues and non-tumor colorectal mucosal tissues, respectively; prognostic role of **(D, E)** SLC2A3 and **(F, G)** PD-L1 expression in CRC samples; **(H)** the relationship between SLC2A3 expression and T, N, M, clinical stage, tumor size, degree of differentiation, vessel invasion, and perineural invasion.

**Figure 8:**
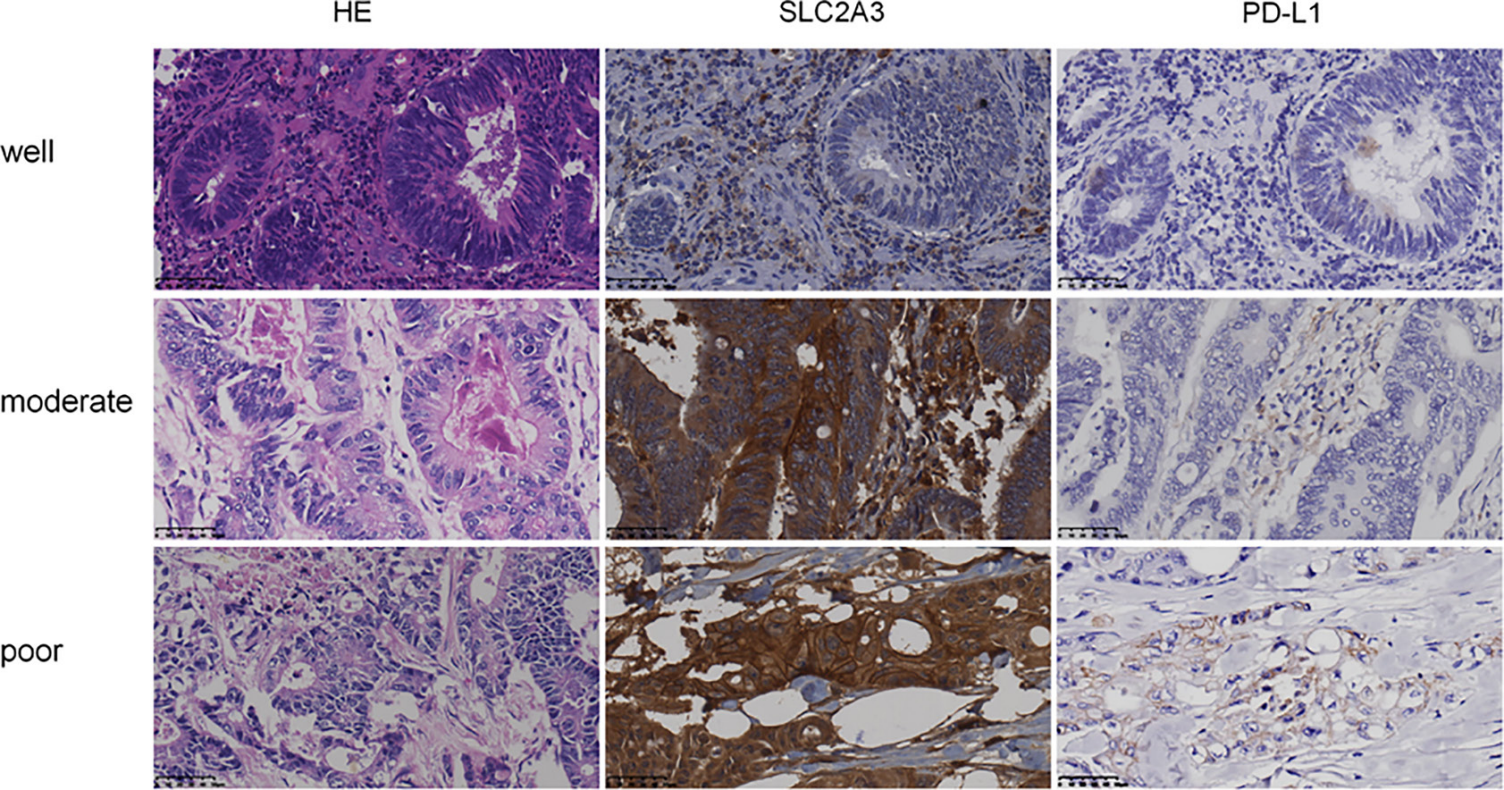
Expression of SLC2A3 and PD-L1 in different differentiated CRC tissues (HE and IHC, original magnification, 400×).

To further explore the expression pattern of SLC2A3 protein in CRC clinical samples, immunohistochemistry (IHC) staining was performed using tissue microarray which contains 174 paired CRC and non-tumor colorectal mucosa tissues. As shown in **Figure 7A**, SLC2A3 signal was localized in the cell membrane, and SLC2A3 protein expression was remarkedly higher in CRC compared with adjacent non-tumor colorectal mucosa tissues (**Figures 7B, C**). In our series, 74% (128/174) CRC showed SLC2A3 positive staining, high and low SLC2A3 expression was 45% (79/174) and 55% (95/174) CRC based on the cutoff value, respectively. High expression of SLC2A3 (**Figures 7D, E**) and PD-L1 (**Figures 7F, G**) was associated with poor overall survival and disease free survival, but it did not reach statistical difference. Furthermore, SLC2A3 expression was significantly associated with perineural invasion (**Figure 7H**). Other features including T, N, M, TNM stage, tumor size, degree of differentiation, vessel invasion was not significantly associated with SLC2A3 expression (**Figure 7H**). While PD-L1 positive staining was found in 22% (39/174)of our CRC samples. Expressions of SLC2A3 and PD-L1 in differently differentiated CRC tissues were shown in **Figure 8**.

**Figure 9:**
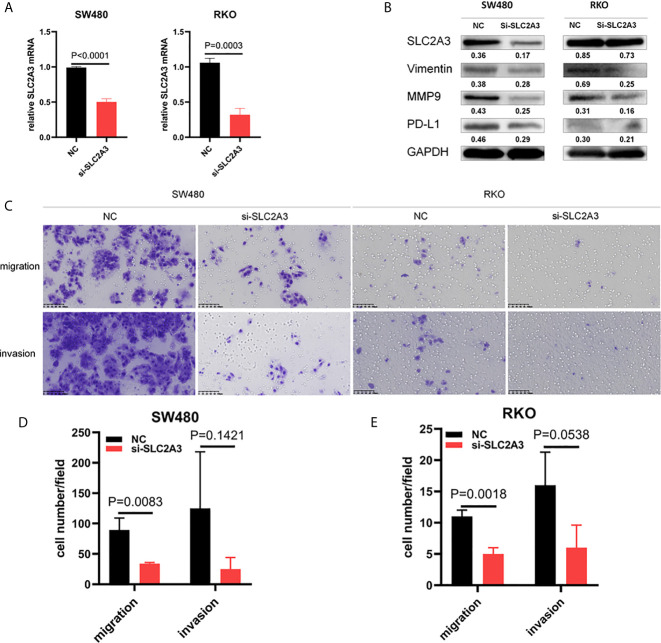
SLC2A3 knockdown inhibited migration in CRC cell lines. **(A)** SLC2A3 mRNA expression decreased in SW480 and RKO transfected with SLC2A3 siRNA, respectively; **(B)** SLC2A3 knockdown inhibited Vimentin, MMP9, and PD-L1 expression in SW480 and RKO by Western blot, respectively; **(C–E)** SLC2A3 knockdown suppressed migration in SW480 and RKO cells, respectively (original magnification, 200×).

To further investigate the role of SLC2A3, SLC2A3 expression was downregulated using siRNA in CRC cell line SW480 and RKO. SLC2A3 mRNA expression level was significantly downregulated compared with the control group (**Figure 9A**). SLC2A3 knockdown inhibited vimentin, MMP9, and PD-L1 expression in SW480 and RKO transfected with SLC2A3 siRNA by Western blot, respectively (**Figure 9B**). Migration and invasion assays showed that SLC2A3 knockdown suppressed migration but not invasion in SW480 and RKO cells compared with the control group, respectively (**Figures 9C–E**).

## Discussion

Recent reports showed that the trend towards CRC is getting younger (27). The 5-year survival rate of CRC was only 20%, indicating that precise therapy for CRC patient needs to be improved (2). In CRC, immune checkpoint therapy have been received approval in 2017 for the subtype of mismatch-repair-deficient (dMMR) or microsatellite instability (MSI-H) (28). Neoantigens accumulation evokes a strong host immune response, which is associated with increased amount of Tumor-Infiltrating Lymphocytes (TILs) and upregulation of immune checkpoint expression (29). An important immune tolerance mechanism is to regulate the response of effector T cells to CTL and stimulate CD4+ T cells to protect tissues from inflammatory damage through immune checkpoints. Among them, many studies were focused on the immune checkpoint molecules CTLA-4 and PD-1 of T cells (30). As a peripheral checkpoint, PD-1 can associated with its ligands PD-L1 or B7-H1/CD274, PDL2 or B7-DC/CD273 to target tumor cells (31). In tumor microenvironment, cancer cells attach to the PD-1 protein of T lymphocytes by the ligand PD-L1, making T cells disable to detect the tumor and tumor cells can escape from the attack by immune system. PD-L1 expression is regulated by complicated processes such as gene transcription, post-transcriptional and post-translational modification. PD-L1 expression in CRC tissues was significantly increased and patients with high PD-L1 expression indicates poor clinical outcome (32). Additionally, Pyo et al. reported that PD-L1 expression in tumor and immune cells of 265 CRC tissues was 25 (9.4%) and 41 (17.7%) respectively. PD-L1 expression in immune cells was significantly associated with clinical features, such as lymph node metastasis and distant metastasis, less lymphatic invasion, lower pT and pTNM stages (33).

Although immunotherapy has shown promising results in CRC, the factors that predict the immune checkpoint response in CRC patients remain unclear. Li et al. have reported that FGFR2 enhances expression of PD-L1 through JAK/SATA3 signaling pathway in CRC (34). Zhang et al. found that Metformin decreased PD-L1 expression *via* activating Hippo signaling pathway in CRC cell lines (35). Using an animal model of CRC, Liu et al. revealed that macrophage-derived CCL5 enhances immune escape of CRC cells through the p65/STAT3-CSN5-PD-L1 pathway (36). These results suggested that the mechanisms of potential immune milieu *via* PD-L1 in CRC.

Chai et al. analyzed the expression of SLC2A family by mining TCGA data and found that SLC2A3 could be used as a marker of CRC in the SLC2A family (37). Kuo et al. have found that SLC2A3 can motivate CRC cell lines’ invasion and stemness *via* activate YAP protein (38). We analyzed the expression level of SLC2A3 in CRC using TCGA database and revealed that high SLC2A3 expression predicted poor prognosis, and the conclusion was verified by two different GSE data. Increasing SLC2A3 expression also indicates more aggressive behavior of CRC, such as higher levels of infiltration and clinic stage. High SLC2A3 expression also indicates the susceptibility of lymph node metastasis, distant metastasis, and MSI-H. The above results suggest that SLC2A3 promotes CRC progression. To clarify the underlying mechanism SLC2A3 in the progression of CRC, GSEA enrichment showed SLC2A3 was involved in EMT pathway and immune response, and SLC2A3 expression was positively correlated with mesenchymal markers and immune reactive-related checkpoints such as PD-L1, PD-L2, LAG3, CTLA4, and TIM3. TIMER website analysis found that SLC2A3 was positively correlated with the infiltration of CD4+ and CD8+T cells in CRC, which was also verified by EPIC. Furthermore, immunohistochemistry staining analysis showed that SLC2A3 expression was significantly higher in CRC compared with that in the adjacent non-tumor colorectal mucosa tissues. Moreover, SLC2A3 expression was significantly associated with perineural invasion of CRC. The result suggests that SLC2A3 promotes progression of CRC.

The underlying mechanism of SLC2A3 in CRC progression remains unclear. Recently, Kuo et al. provided evidence that SLC2A3 can promote metastasis property of brain cancer cell and cAMP-response element binding protein (CREB) can directly regulate SLC2A3 expression (9). SLC2A3 can accelerate AML development through impaired vitamin C uptake and diminish TET2 restoration (39). SLC2A3-STAT3-SLC2A3 feedback loop may strengthen phosphorylation of the STAT3 signaling pathway and SLC2A3 may involve in gastric cancer immune response by promote M2 subtype transition of macrophage infiltration (40). Consistent with these results, our study showed that SLC2A3 knockdown significantly suppressed migration ability in CRC cell line SW480 and RKO transfected with SLC2A3 siRNA. Further study showed that SLC2A3 promoted the progression of CRC through EMT. Moreover, the expression of PD-L1 also decreased with the downregulation of SLC2A3 expression, indicating that SLC2A3 may participate in the immune response of CRC through PD-L1. The results suggest that SLC2A3 promote CRC progression by regulating EMT and immune response. The underlying molecular mechanism of SLC2A3 regulating EMT and immune markers including PD-L1 in development and progression of CRC needs further study in the future.

There are some limitations in our study. First, whether SLC2A3 regulates EMT and immune response directly or indirectly and its molecular mechanism needs further study. Second, migration and invasion function *in vitro* still needs to be verified by *in vivo* animal metastasis model. In summary, our study shows that SLC2A3 is involved in the progression of CRC through EMT and immune response, and SLC2A3 could be used as the prognosis marker of CRC and a novel candidate target for CRC treatment.

## Data Availability Statement

The original contributions presented in the study are included in the article/supplementary material. Further inquiries can be directed to the corresponding authors.

## Author Contributions

HG, JL, and JD performed experiment and data analysis. LC, HL, and TZ collected data. FZ and YD provided the tissue specimen and staining. HS performed the data analysis. AH designed and revised the manuscript. All authors contributed to the article and approved the submitted version.

## Funding

This work was supported by the National Natural Science Foundation of China (Grant Nos. : 81472251, 81272636).

## Conflict of Interest

The authors declare that the research was conducted in the absence of any commercial or financial relationships that could be construed as a potential conflict of interest.
